# A Correlational Analysis between the Rate of Force Development among the Arm Stroke, the Leg Kick, the Full Stroke and Short Distance Front Crawl Speed in Highly Trained Swimmers

**DOI:** 10.5114/jhk/204780

**Published:** 2025-09-23

**Authors:** Xiaotong Chen, Yongshen Lu, Bowei Zhang, Yupeng Shen

**Affiliations:** 1 School of Sports Science, South China Normal University, Guangzhou, China.; 2 Sports Academy, Guangzhou College of Commerce, Guangzhou, China.; 3 Sports Department, Shenzhen University, Shenzhen, China.

**Keywords:** force-time curve, propulsive force, tethered swimming, neuromuscular performance, sex differences

## Abstract

This study aimed to analyze sex differences in the rate of force development (RFD) among the arm stroke only (As), the leg kick only (Lk), and the full stroke (Fs) through the 30-s front crawl tethered-swimming (TS) test, and to discuss the correlation between RFD variables and short-distance front crawl speed in the force-time (F-t) curve across different sexes. Sixteen male and fourteen female highly trained swimmers (age: 19.7 ± 3.27 years) performed the TS test under three conditions: As, Lk, Fs, as well as a 25-m front crawl test. The As, Lk, Fs of the peak RFD per 5 s (RFD_ave_), and maximum value of the peak RFD every 5 s (RFD_max_) had highly significant correlation with V25, V50, and V100 (V25−V100). Notably, the Fs of the male RFD and the As of the female RFD showed particularly strong correlations with V25−V100. It was also observed that there were sex-specific disparities in the RFD indices associated with the As, Lk, and Fs phases of swimming. The findings indicate that the RFD within the F-t curve is a valuable variable for assessing the capacity to generate propulsive force in water. Sex factors affect the relationship between RFD_ave_, RFD_max_, and short-distance front crawl speed in swimming. Coaches should fully consider sex differences when training for swimming propulsive force.

## Introduction

Propulsive force is recognized as a critical determinant of speed changes within the context of swimming performance (Barbosa et al., 2013; Santos et al., 2024). Owing to the complexities introduced by fluid dynamics, direct measurement of the propulsive force generated by a swimmer is not feasible. Instead, researchers rely on indirect methodologies to estimate these forces. Among these indirect approaches, tethered swimming (TS) has been established as both an effective and reliable technique for assessing propulsive force variations across the four competitive swimming strokes, at different phases of the stroke cycle (Morouço et al., 2011) and for different body segments (Amaro et al., 2014; Magel, 1970; Nagle et al., 2016). Despite the fact that absolute values such as the peak (Santos et al., 2016), mean (Amaro et al., 2014; Loturco et al., 2015), and impulse (Santos et al., 2016) of the force-time (F-t) curve (Soncin et al., 2017) during TS tests serve as ideal variables for predicting maximal swimming speed, the brief duration over which force is generated during a complete swimming stroke cycle renders these absolute values deficient in their consideration of the temporal aspect.

In competitive swimming, highly trained athletes should enhance their swimming speed by improving the ability to rapidly release force within a unit of time (Andrade et al., 2018). This ability is contingent upon the manifestation of the F-t variation, namely the rate of force development (RFD). Force, work, and power are variables frequently employed to characterize neuromuscular performance (Ide et al., 2023). The RFD is the slope of the F-t curve during muscle contraction over a specified time frame, particularly indicative of the force generation in the initial phase of muscle contraction (Aagaard et al., 2002). This measure offers a more precise quantitative evaluation of neuromuscular performance during explosive contractions. During rapid explosive movements, highly trained athletes exhibit a higher RFD compared to well-trained athletes, and elevated RFD values are associated with enhanced athletic performance (Maffiuletti et al., 2016; Slawinski et al., 2010). Short-distance swimming is categorized as a rapid explosive movement. Presently, in the methodologies employing TS to measure propulsive force, there is a predominant focus on assessing the correlation between changes in absolute values and swimming speed. However, the relationship between the aquatic RFD and short-distance swimming speed remains to be elucidated.

Swimming necessitates the coordinated effort of the limbs to generate propulsive force, essential for propelling the body forward in water. Given the distinct structural and functional characteristics of the human arms and legs (Wannier et al., 2001), an imbalance exists in the propulsive forces produced by arm strokes only (As) and leg kicks only (Lk). It is generally agreed that the arms provide more than 85% of the total thrust in the crawl stroke (Hollander et al., 1987; Rackham, 1975; Toussaint and Beek, 1992). Nonetheless, the propulsive force from the legs plays a crucial role in controlling the body position in water (Gourgoulis et al., 2014), thus sustaining high-speed swimming. Research has indicated that the combined contributions of propulsive force from the isolated arm and leg actions do not equate to the total propulsive force generated through full limb coordination (Deschodt et al., 1999). This suggests that there may be inherent differences in the relative contributions of the As and the Lk to overall propulsive force, yet a comprehensive study on this topic remains absent.

In the realm of competitive swimming, male swimmers consistently achieve faster speeds than their female counterparts, a disparity that reflects the distinct physiological attributes between sexes. Typically, males exhibit greater muscle strength and an enhanced capacity for force production (Hannah et al., 2012). However, when accounting for peak force and variations in muscle mass or volume, the male advantage may be less pronounced or may not exist at all (Guignard et al., 2019). Presently, the methodologies employing TS to measure propulsive force predominantly focus on assessing the correlation between changes in absolute values and swimming speed. The relationship between the aquatic RFD during As, Lk and full stroke (Fs) movements of highly trained male and female athletes with short-distance swimming speed warrants further investigation. Gaining insights into the RFD associated with As, Lk and Fs movements is crucial for coaches to provide tailored guidance to male and female athletes, to develop training programs scientifically, and to enhance the explosive power in short-distance swimming.

This study employed the 30-s front crawl TS to explore the relationship between RFD variables for the As, the Lk, and the Fs, as depicted in the F-t curve and short-distance front crawl swimming speed across different sexes. The investigation also aimed to scrutinize potential sex disparities in these RFD variables. The findings offer guidance for assessing the swimming RFD, which is beneficial for coaches to further understand the ability of swimmers of different sexes to generate force in the water.

## Methods

### Participants

Thirty highly trained swimmers (16 males and 14 females: age = 19.7 ± 3.27 years, body height = 176.83 ± 8.45 cm, body mass = 68.56 ± 10.73 kg) participated in this study. The personal best times for males in the current 50-m event were 24.7 ± 1.24 s, and for the 100-m crawl were 53.75 ± 2.63 s. Corresponding times for the females were 27.46 ± 1.25 s for the 50-m event, and 60.51 ± 2.98 s for the 100-m front crawl. These times were equivalent to 81.86% and 85.15% of the male's world records, and 82.91% and 82.99% of the female's world records, respectively (McKay et al., 2022). Prior to the experiment, highly trained swimmers were required to fulfill the following criteria: (i) had the professional swim training background of more than 10 years, (ii) performed extreme-intensity swimming training at least twice a week, with approximately 16 hours of training per week, (iii) had no musculoskeletal injuries in the past six months. This study obtained informed consent from all participants, and all procedures were reviewed and approved by the ethics committee of the South China Normal University, Guangzhou, China (protocol code: SCNU-SPT-2022-101; approval date: 22 November 2022).

### Design and Procedures

#### Design

This research utilized a repeated measures design, administering a comprehensive set of swimming performance assessments to athletes. Highly trained swimmers performed a 25-m front crawl test and a 30-s front crawl TS test under three conditions: the As, the Lk, and the Fs. To ensure uniformity in testing conditions, all evaluations were conducted in a temperature-controlled 50-m indoor pool, and each condition was completed during the same morning or afternoon training session.

The TS test has been proven to be an effective method for assessing anaerobic performance and propulsive force in the water, with high reliability and validity (Amaro et al., 2014; Morouço et al., 2014), and it has also been used to evaluate the propulsive force of the front crawl stroke, the leg kick, and coordination (Bartolomeu et al., 2024; Morouco et al., 2015). Propulsive forces in this study were measured by a Swimsportec swimming force measurement device (Otto Otto GmbH, Germany). Throughout the TS test, a belt was tied around the athlete's waist, and this belt was simultaneously connected to a steel cable, which was attached to the force measurement device. The device was fixed to the starting block of the swimming lane and aligned as horizontally as possible to minimize interference with the swimmer's movement. When the athlete started swimming, the tension in the steel cable activated the force sensor, capturing real-time mechanical data at a sampling rate of 31 Hz. Concurrently, the SwimAnalyzer software recorded the F-t data for the entire 30-s TS test, providing precise data for subsequent analysis (Morouço et al., 2024).

### Procedures

On the same day, participants undertook two of the four tests—maximum speed 25-m front crawl swimming, the As, the Lk and the Fs 30-s front crawl TS test—during both morning and afternoon training sessions, in randomized order. Prior to each test session, participants completed an unsupervised warm-up of moderate to low intensity for over 20 min, followed by a 10-min rest period before commencing the formal experiment. An active rest period of 30 min was allowed between each test trial. The rest interval between the trials was 6 min to achieve total recovery (Hancock et al., 2015). Previous studies have proven that in the depleted muscle 100% of the ATP and PCr are restored within 3–5 min (Bogdanis et al., 1998). Consequently, the 30-min active rest period between tests was considered to have no significant impact on the test outcomes.

### Measures

#### The 25-m Front Crawl Test

Participants were required to initiate the swim with a push-off from the pool wall, refraining from performing underwater dolphin kicks to avoid the impact of starts and glides on the speed test. Records were collected by experimenters who had many years of swimming training experience and had previously supervised training. A stopwatch (CASIO, HS-70W-1JH, Japan) was used to record the swimming time from the head (side edge of the swim cap) to the 5-m mark to the head to the 20-m mark (Soncin et al., 2017), which was marked with a reflective waterproof sticker on the side of the swimming pool prior to the experiment (Hay et al., 1983), which made it easier for the research staff to record the time more accurately. Manual recording of swim-in times and stroke counts was more common and feasible for swim coaches' daily training.

#### The 30-s Front Crawl Tethered-Swimming Test

**Figure 1:**
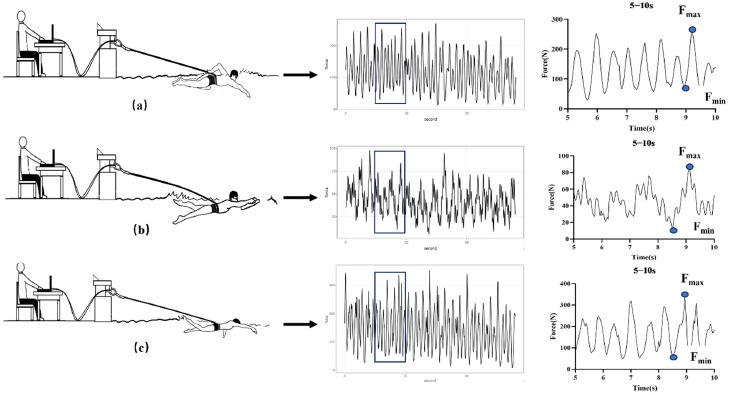
Illustration of the As (a), Lk (b), Fs (c) tethered swimming test.

Participants performed the 30-s TS test under three conditions in a random sequence: the As, the Lk, and the Fs (Figure 1). Before the test, a 5-s simulated TS test was conducted to familiarize participants with the experimental process, and with the steel cable of the force measuring device secured around the participant's waist using a belt. For the TS of the As, participants were required to hold an A-shaped float between their thighs, keeping their legs immobile; for the TS of the Lk, participants held an A-shaped float with their hands, keeping their arm still; for the TS of the Fs, participants were expected to use synchronized hand and leg movements to complete the swim. Following the simulated test, participants engaged in active rest for 5 min before commencing the formal experiment. Before the start signal, participants assumed a stationary prone position in the water with the steel cable nearly taut. Upon the start signal, participants adopted their customary breathing pattern used in front crawl swimming and exerted a full 30-s sprint until the researcher signaled to stop. Throughout the experiment, participants were consistently verbally encouraged to swim at maximum speed.

### Data Analysis

The average speed from the 25-m front crawl maximal speed swim test (V25), along with the average speed of the personal best performances in the 50-m and 100-m front crawl (V50, V100) and the sprint times (T50, T100) were selected as kinematic variables. Personal best performances were the best swim times over short distances obtained during the experiment ± 90 days, during the summer and winter seasons when athletes were at their competitive peak (Haan et al., 2024; Maglischo, 2003). The F-t curve variables, such as maximum force (F_max_), minimum force (F_min_), mean force (F_mean_), the average rate of force development (RFD_ave_), the maximum RFD (RFD_max_) and impulse were selected as dynamical variables. Studies demonstrated that during TS (Amaro et al., 2014; Loturco et al., 2015), the F-t curve variables including F_max_ (ICC = 0.942, CV = 1.27%), F_mean_ (ICC = 0.955, CV = 3.92%), impulse (ICC = 0.99, CV = 1.14%), and the RFD (ICC = 0.72, CV = 6.9%) had high levels of reliability and consistency. While limited research examined the reliability and consistency of F_min_ and *Df* in the context of TS, these variables, which are derived from the F-t curve, are recognized as common dynamical variables.

The force variables acquired during TS can be calculated employing the equation established by Morouço et al. (2018). The RFD is primarily determined using the methodologies outlined by Hernández-Davó and Sabido (2014) and Loturco et al. (2015) for both the peak RFD and the time interval RFD. *Df* and RFD variables were calculated using equations 1 and 2 as follows:


$$Df=\frac{\sqrt{\frac{\sum {}_{i}{\left(f_{i}-\bar{f}\right)}^{2}F_{i}}{n}}}{\frac{\sum {}_{i}f_{i}\cdot F_{i}}{n}}\cdot 100$$

where *Df* was the intracyclic variation of the horizontal speed of the hip, $\bar{f}$ the mean swimming force, *f_i_* the instant swimming force (31Hz), *F_i_* the acquisition frequency, and *n* the number of observations.

RFD (N•s^−1^) = peak force (N) / time to peak force (s) (2)

where RFD was the F-t curve recorded during explosive active contraction. From equation 2, the RFD was calculated in six 5-s intervals of 0–5 s, 5−10 s, 10−15 s, 15−20 s, 20−25 s and 25−30 s (Figure 1). The average and the maximum of the RFD in six 5-s intervals were selected as RFD_ave_ and RFD_max_ for TS.

### Statistical Analysis

The StatsModels library in Python (v.3.13.0, Netherlands) was used to conduct statistical power analysis and to evaluate the sample size needed for the experiment. Based on a two-factor repeated-measures ANOVA study design using Cohen's *d* effect size (*d* = 0.6), significance level (α = 0.05), and expected statistical efficacy (power = 0.8), the required sample size was calculated to be 23 participants per group. Further analysis was conducted on the sample size and statistical power in the post-hoc multiple comparisons. For within-group comparisons (Lk vs. As vs. Fs), a minimum of 14 subjects per group was required to meet the criteria of effect size = 0.8, significance level = 0.05, and statistical power = 0.8. For between-group comparisons (male vs. female), the calculated statistical power based on the current sample size was 0.59 (effect size = 0.8, significance level = 0.05). This indicates that the statistical power for between-group comparisons might be insufficient, and the results should be interpreted with caution.

The normality of variable distribution was assessed using the Kolmogorov-Smirnov test for normality. Descriptive statistics were employed to report the physical characteristics, speed achievements, and force value data of the research subjects (M ± SD). A two-factor repeated measures analysis of variance (ANOVA) was utilized to analyze differences in RFD variables between sexes and limbs, with F values, *p* values and η^2^ values reported to ascertain the significance of main effects. The magnitude of effects was classified based on the absolute value of Cohen's *d* (|*d*|), where a value of 0.2 ≤ |*d*| ≤ 0.5 indicated a small effect, 0.5 < |*d*| ≤ 0.8 signified a medium effect, and |*d*| > 0.8 denoted a large effect. The Pearson correlation coefficient (*r*) was utilized to evaluate the relationship between RFD variables and short-distance speed, with correlation thresholds for very low, low, moderate, high, and very high defined as 0, 0.2, 0.4, 0.6, and 0.8, respectively (Cohen, 2013). All statistical analyses were performed using JASP 15.0, with statistical significance set at *p* < 0.05.

## Results

### Correlational Analysis between the Rate of Force Development in the Lower & Upper Limbs and Short-Distance Front Crawl Swimming Speed

Tables 1 and 2 show that significant correlations existed between the RFD_ave_, the RFD_max_ of the As, Lk, Fs and V25–V100 and there were differences in sex. For male athletes, V25–V100 showed a high positive correlation with the RFD_ave_, and the RFD_max_ of the Fs (*r* = 0.63–0.78, *p* < 0.01); for female athletes, V50–V100 demonstrated a high positive correlation with the RFD_ave_, and the RFD_max_ of the As (*r* = 0.54–0.74, *p* < 0.05). A moderate positive correlation was observed between V25 and the RFD_ave_ of the As for male athletes, and between V100 and the RFD_ave_ of the Fs for female athletes (*r* = 0.5–0.57, *p* < 0.05).

**Table 1:** Correlation coefficients of RFD variables with V25−V100 for 30-s front crawl tethered-swimming.

Speed	Variable	Lk	As	Fs
V25	RFD_ave_	0.42^*^	0.75^**^	0.73^**^
	RFD_max_	0.36^*^	0.68^**^	0.64^**^
V50	RFD_ave_	0.43^*^	0.73^**^	0.79^**^
	RFD_max_	0.4^*^	0.68^**^	0.69^**^
V100	RFD_ave_	0.45^*^	0.79^**^	0.84^**^
	RFD_max_	0.37^*^	0.76^**^	0.78^**^

* p < 0.05, ^**^ p < 0.01; V25: speed in the 25-m front crawl test; V50: speed in the 50-m front crawl test; V100: speed in the 100-m front crawl test; RFD_ave_: mean value of the peak RFD per 5 s; RFD_max_: maximum value of the peak RFD every 5 s; Lk = Leg kick, As = Arm stroke, Fs = Full stroke

**Table 2:** Correlation coefficients of RFD variables with V25−V100 for 30-s front crawl tethered-swimming of different sexes.

Speed	Variable	Male			Female		
		Lk	As	Fs	Lk	As	Fs
V25	RFD_ave_	0.07	0.51^*^	0.65^**^	0.25	0.57^*^	0.25
	RFD_max_	−0.09	0.39	0.63^**^	0.33	0.54^*^	−0.07
V50	RFD_ave_	−0.13	0.3	0.65^**^	0.47	0.7^**^	0.52
	RFD_max_	−0.12	0.26	0.66^**^	0.45	0.66^*^	−0.03
V100	RFD_ave_	−0.13	0.46	0.78^**^	0.52	0.74^**^	0.57^*^
	RFD_max_	−0.21	0.44	0.77^**^	0.44	0.73^**^	0.26

^*^ p < 0.05, ^**^ p < 0.01; V25: speed in the 25-m front crawl test; V50: speed in the 50-m front crawl test; V100: speed in the 100-m front crawl test; RFD_ave_: mean value of the peak RFD per 5 s; RFD_max_: maximum value of the peak RFD every 5 s; Lk = Leg kick, As = Arm stroke, Fs = Full stroke

### Analysis of Sex Differences in the Rate of Force Development for the Lower & Upper Limbs

Table 3 presents the results from the repeated measures analysis of variance (ANOVA) based upon the F-t curve model. In the female RFD_ave_ variable, the Fs was significantly higher than the Lk [MD = −363.85, 95%CI = (–655.94, –71.75), *p* = 0.01] and the As was also significantly higher than the Lk [MD = –269.44, 95%CI = (–561.53, 22.66), *p* = 0.05]; in the female RFD_max_ variable, the Fs was significantly higher than the Lk [MD = –456.24, 95%CI = (–611.7, –300.79), *p* < 0.01] and the As [MD = –127.1, 95%CI = (–282.51, 28.39), *p* = 0.05], and the As was significantly higher than the Lk [MD = –329.18, 95%CI = (–484.64, –173.73), *p* < 0.01].

**Table 3:** Analysis of sex differences in the RFD in the lower and upper limbs.

RFD variable	Sex	Lk (M ± SD)	As (M ± SD)	Fs (M ± SD)	Result of the repeated measures ANOVA					
					Effect of limbs			Limbs × sex		
					F	*p*	η^2^	F	*p*	η^2^
RFD_ave_ (N•s^−1^)	F	79 ± 33.3	348.4 ± 86.9	442.8 ± 70.1	22.9	< 0.01	0.45	21.3	< 0.01	0.43
	M	240.4 ± 514.4	594 ± 146.9	728.7 ± 209.4						
RFD_max_ (N•s^−1^)	F	117.4 ± 58.8	446.5 ± 125.9	573.6 ± 134.3	157.1	< 0.01	0.85	26.7	< 0.01	0.49
	M	162.5 ± 72	768.5 ± 228.2	866.4 ± 231.2						

F: female; M: male; RFD_ave_: mean value of the peak RFD per 5 s; RFD_max_: maximum value of the peak RFD every 5 s; Lk = Leg kick, As = Arm stroke, Fs = Full stroke

In contrast, the Fs was significantly higher than the Lk [MD = –488.3, 95%CI = (–761.53, –215.07), *p* < 0.01] and the As was also significantly higher than the Lk [MD = –353.62, 95%CI = (–626.86, –80.39), *p* = 0.00] in the male's RFD_ave_ variables; in the male's RFD_max_ variables, the Fs was significantly higher than the Lk [MD = –703.96, 95%CI = (–849.37, –558.55), *p* < 0.01] and the As was significantly higher than the Lk [MD = –606.01, 95%CI = (–751.43, –460.6), *p* < 0.01].

Statistically significant differences of minor magnitude were observed in the RFD_ave_ among the Fs, the As and the Lk (F = 22.9, η^2^ = 0.45, *p* < 0.01), while the RFD_max_ variable demonstrated statistically significant differences of major magnitude (F = 157.11, η^2^ = 0.85, *p* < 0.01). Furthermore, the sex factor exhibited significant differences of minor magnitude in both the RFD_ave_ (F = 21.3, η^2^ = 0.43, *p* < 0.01) and the RFD_max_ (F = 26.65, η^2^ = 0.49, *p* < 0.01) across the Fs, the As and the Lk.

### Correlation Analysis of the RFD and Chosen Variables of the F-t Curve in the Lower and Upper Limbs

Table 4 shows that for male athletes, the RFD_ave_ and the RFD_max_ of the As and the Fs exhibited a moderate to very high positive correlation with F_max_, F_mean_ and impulse variables (*r* = 0.56–0.9, *p* < 0.05); the RFD_ave_ of the Lk demonstrated a moderate positive correlation with *Df* (*r* = 0.51, *p* < 0.05). For female athletes, the RFD_ave_ and the RFD_max_ of the As with F_max_, and the RFD_ave_ of the Lk with *Df* exhibited a moderate to high positive correlation (*r* = 0.58–0.73, *p* < 0.05).

**Table 4:** Correlation coefficients of the RFD variables and the F-t variables.

Speed	Variable	Male			Female		
		Lk	As	Fs	Lk	As	Fs
F_max_ (N)	RFD_ave_	0.36	0.84^**^	0.9^**^	−0.13	0.73^**^	0.47
	RFD_max_	0.4	0.9^**^	0.82^**^	−0.11	0.58^*^	0.53
F_min_ (N)	RFD_ave_	−0.27	0.18	0.12	0.00	0.2	−0.09
	RFD_max_	−0.01	0.01	0.28	−0.08	0.2	−0.2
F_mean_ (N)	RFD_ave_	0.15	0.61^*^	0.84^**^	−0.27	0.51	0.19
	RFD_max_	0.31	0.56^*^	0.78^**^	−0.26	0.35	−0.21
*Df* (%)	RFD_ave_	0.51^*^	−0.18	−0.27	0.61^*^	−0.16	0.13
	RFD_max_	0.21	−0.01	−0.37	0.49	−0.05	0.48
Impulse (N•s)	RFD_ave_	0.15	0.61^*^	0.84^**^	−0.27	0.51	0.19
	RFD_max_	0.31	0.56^*^	0.78^**^	−0.26	0.35	−0.21

^*^ p < 0.05, ^**^ p < 0.01; F: Female; M: Male; RFD_ave_: mean value of the peak RFD per 5 s; RFD_max_: maximum value of the peak RFD every 5 s; Lk = Leg kick, As = Arm stroke, Fs = Full stroke; F_max_: maximum force, F_min_: minimum force, F_mean_: mean force, Df: intracyclic force variation

## Discussion

In this study, we found that RFD variables (RFD_ave_ and RFD_max_) were significantly correlated with short-distance swimming speeds (25 m, 50 m, and 100 m) through the 30-s TS test. When considering the correlation between RFD variables and traditional absolute value variables of force, we also found that the RFD of the As and the RFD of the Fs in males correlated with the maximum, mean, and impulse of force; the RFD of the As in females correlated with the maximum force, and the RFD of the Lk correlated with the fluctuation rate of force. This indicates that the RFD at different stages on the F-t curve can effectively reflect the changes in speed during short-distance swimming and is also an important variable for evaluating the performance of swimming propulsion force. Swimming is a dynamic movement process, and speed changes depend not only on the maximum force generated by muscles, but also on the efficiency of applying these forces underwater and converting them into propulsive force (Sharp et al., 1982). In terms of assessment methods, traditional TS focuses more on absolute values of force, such as maximum or average propulsive force, but these variables fail to fully capture the dynamic changes in force (Morouço et al., 2018; Santos et al., 2016). Propulsive force is not only an absolute value, but should also include a time dimension. In fact, the product of force and speed (power) has been found to be highly correlated with short-distance front crawl performance, and power can fully reflect changes in propulsive force (Haff et al., 1997). However, power, as a scalar, does not provide a complete picture of the process by which a muscle generates force in the shortest possible time. In contrast, neuromuscular performance is a vector quantity that encompasses both the magnitude of force as well as the direction and time dimensions of force production. Therefore, the best measure of neuromuscular performance is the RFD, as it reflects the ability of muscles to generate maximum force in the shortest time (Maffiuletti et al., 2016), thus providing a more accurate assessment of an athlete's performance during high-speed swimming. Although studies have examined the variation of peak and average force on the F-t curve in TS, this study further demonstrates that the average and peak RFD at different time intervals is a more complete analysis of propulsive force. This time-based approach to RFD assessment allows to analyze muscle force output during swimming more accurately (Bartolomeu et al., 2024; Haff et al., 2015) and provides a new perspective and variables for exploring the variation in swimming propulsion, as well as the relationship between the ability to generate force in water and speed performance.

This study highlighted significant sex-based disparities in RFD_ave_ and RFD_max_ variables for the As, the Lk and the Fs. Specifically, in males, V25–V100 variables were strongly correlated with the RFD of the Fs, while in females, these speeds showed a predominant correlation with the RFD of the As. Moreover, males exhibited a higher RFD in the Fs compared to the As and the Lk, whereas females demonstrated a superior RFD in the As and the Lk relative to the Fs. These differences extended to propulsive force, where males generally displayed an advantage for the As and the Lk, evidenced by their higher peak and average propulsive forces. This advantage may be attributed to greater absolute strength and muscle power in males (Bishop et al., 1989; Guignard et al., 2019; Morouco et al., 2015; Oliveira et al., 2021). Furthermore, whole-body muscular coordination is essential for effective propulsion during swimming (Toussaint and Beek, 1992). Optimizing the coordination of the As and the Lk is particularly critical, as studies have shown that vortices generated by arm movements can enhance leg propulsion, thereby increasing swimming speed (Cohen et al., 2015; Hochstein and Blickhan, 2011; Millet et al., 2002). For males, their longer limbs, along with a greater stroke length and rate, contribute to higher propulsion efficiency in the Fs (Nevill et al., 2020). In contrast, females experience reduced frontal drag with their arms, but must compensate for shorter stroke lengths by increasing their kicking frequency, which can lead to higher energy consumption (Gatta et al., 2012; Schnitzler et al., 2008; Zamparo et al., 1996). Notably, female swimmers exhibit better arm coordination, which likely enhances mechanical power output during arm strokes (Schnitzler et al., 2008). The RFD, as a critical indicator of mechanical power output (Aagaard et al., 2002), helps explain why females showed stronger correlations between the RFD of the As and speed. Conversely, males rely more on peak force of the As for speed, whereas females depend more on average force of the Fs (Morouco et al., 2015). However, this finding differed from previous studies, which may be due to differences in the methods of propulsive force analysis. While earlier research primarily focused on peak and mean instantaneous forces, this study analyzed the slope of the F-t curve (RFD variable) during a 30-s TS test. Specifically, the RFD_ave_ and the RFD_max_ represent the mean and maximum RFD across six 5-s intervals, offering a more detailed description of the force-rate dynamics over time (Sleivert and Wenger, 1994).

The present study also found a low correlation between the RFD of the Lk and the speed range V25–V100; however, when analyzed by sex, there was no correlation between the RFD of the Lk and V25–V100. This may be due to the limitations of the front crawl swimming leg kick pattern when using the equipment for the TS test. In a previous study on leg propulsion, it was found that leg propulsion could significantly increase the maximum speed of Fs by 10% (Deschodt et al., 1999). In contrast, in front crawl sprinting, leg kicking reduces the pressure drag acting on the trunk and reduces the energy output of the propulsive force of arms. This potentially suggests that the RFD of the Lk over a shorter period of time may be critical for maintaining higher swimming speeds and keeping the body position balanced. Furthermore, research on the RFD of the Lk should not be overlooked.

This study has some limitations in the selection of subjects and testing of swimming strokes. All the subjects were highly trained swimmers with an average age of adulthood, and they differed from youth athletes and elite swimmers in terms of swimming technique and muscle strength (Scott et al., 2024). Future research could further analyze the RFD performance of butterfly, breaststroke, and backstroke swimming styles, because in the present study we only evaluated the RFD of the front crawl, but did not compare the sex differences in the correlation between the RFD of arms, legs, and coordination and short-distance speed in the four swimming strokes. Additionally, considering the unbalanced selection of male and female sample sizes in this study, caution should be exercised when discussing the sex differences in RFD variables, and the sample size should be expanded in future research.

## Conclusions

Using the RFD variable on the F-t curve, we can analyze the ability to generate force in the water. This provides a new perspective for assessing the neuromuscular performance of swimmers, as well as their ability to generate power, perform work, and transfer power in swimming. Additionally, we found that sex factors influenced the relationship between the RFD of the As, the Lk and the Fs with short-distance speed in the water. Short-distance speed had a high correlation with the RFD of the Fs in males and the RFD of the As in females. When assessing propulsive forces in the water, full consideration should be given to sex and the differences between the lower and upper limbs.
